# Primary Gastrointestinal Diffuse Large B-cell Lymphoma

**DOI:** 10.7759/cureus.3258

**Published:** 2018-09-05

**Authors:** Hanan T Lodhi, Qulsoom Hussain, Ahmed Munir, Fahad Zafar, Zarak H Khan

**Affiliations:** 1 Infectious Disease, University of Nebraska Medical Center, Omaha, USA; 2 Medicine, Shifa International Hospital, Islamabad, PAK; 3 Medicine, Services Institute of Medical Sciences, Lahore, PAK; 4 Internal Medicine, King Edward Medical University, Lahore, PAK; 5 Internal Medicine, St. Mary Mercy Hospital, Livonia, USA

**Keywords:** diffuse large b cell lymphoma, gastrointestinal lymphoma, non-hodgkin cell lymphoma, human immunodeficiency virus (hiv), jejunal lymphoma

## Abstract

Primary gastrointestinal lymphoma is a rare neoplasm that accounts for less than 5% of all gastrointestinal malignancies. We present a case of a 37-year-old woman positive for human immunodeficiency virus who presented with abdominal pain and vomiting for three months. She underwent endoscopic biopsy and was found to have high-grade diffuse large B-cell lymphoma in the jejunum. This report discusses her treatments and includes a brief literature review highlighting the rarity of this entity, the etiological agents implicated in its pathogenesis, and the lack of specific guidelines for treatment.

## Introduction

Most cases of gastrointestinal lymphoma are due to widespread nodal disease. Primary gastrointestinal tract lymphoma is a rare entity, accounting for 1%-4% of all gastrointestinal malignancies, and its occurrence primarily in the small intestine is even more uncommon [1].

The overall incidence of diffuse large B-cell lymphoma (DLBCL) in the Midwestern region of the United States from 1992 to 2001 was approximately seven cases per 100,000 persons per year [2]. There have not been studies to decipher the incidence of DLBCL in the gastrointestinal tract. However, a study done in 1,010 patients in China found 58% of primary gastrointestinal lymphomas to be DLBCL, and, of the total intestinal lymphomas, 53% were DLBCL [3].

Endoscopic evaluation is garnering wider acceptance for use in the diagnosis of gastrointestinal lymphoma. Surgery was the preferred treatment for primary gastrointestinal lymphoma, but newer research supports chemotherapy as the first-line treatment, especially in a highly aggressive lymphoma such as DLBCL. Our case represents a unique presentation of DLBCL as abdominal pain in a 37-year-old woman with human immunodeficiency virus (HIV).

## Case presentation

A 37-year-old woman with a history significant for HIV/acquired immunodeficiency syndrome (AIDS) (treated via anti-retroviral therapy), and epilepsy (treated via anti-epileptic meeldications) presented with abdominal pain ongoing for three months associated with nausea and vomiting. The pain was diffuse, radiating to her back, and it limited her oral intake. She reported night sweats and chills but did not recall exposure to any people with signs of illness. Clinical laboratory tests were performed, and abdominal computed tomography (CT) scan was ordered.

Her most recent CD4 count was 37 cells/mm^3^ (reference range: 500–1,500 cells/mm^3^). The CT scan of her abdomen and pelvis showed diffuse irregular small bowel wall thickening and submucosal edema along with retroperitoneal and diffuse mesenteric lymphadenopathy. Based on her history and the radiology findings, the patient received an endoscopy. The endoscopy revealed lymphoid nodules in the gastric body.

**Figure 1:**
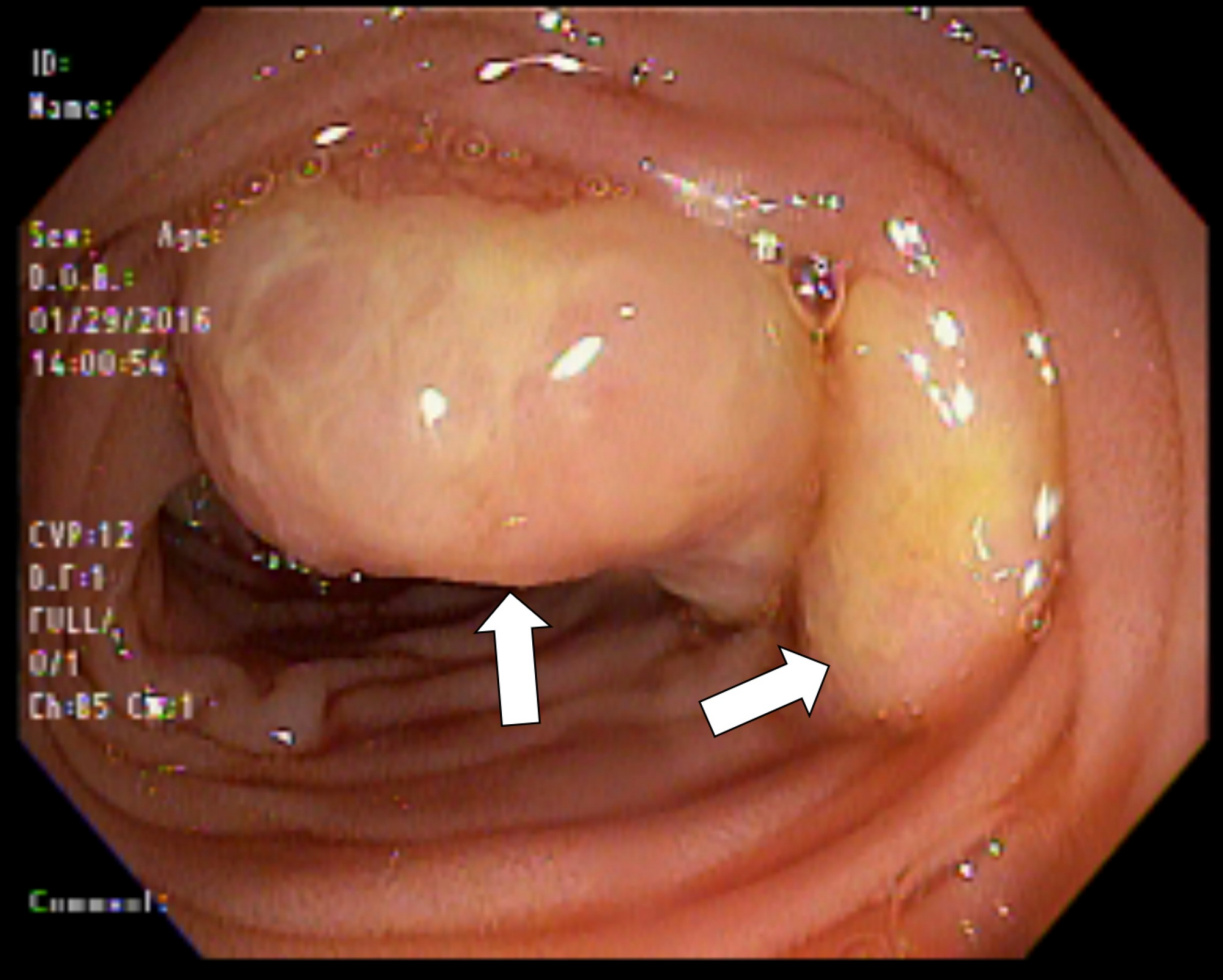
Nodules seen endoscopically in duodenum.

**Figure 2:**
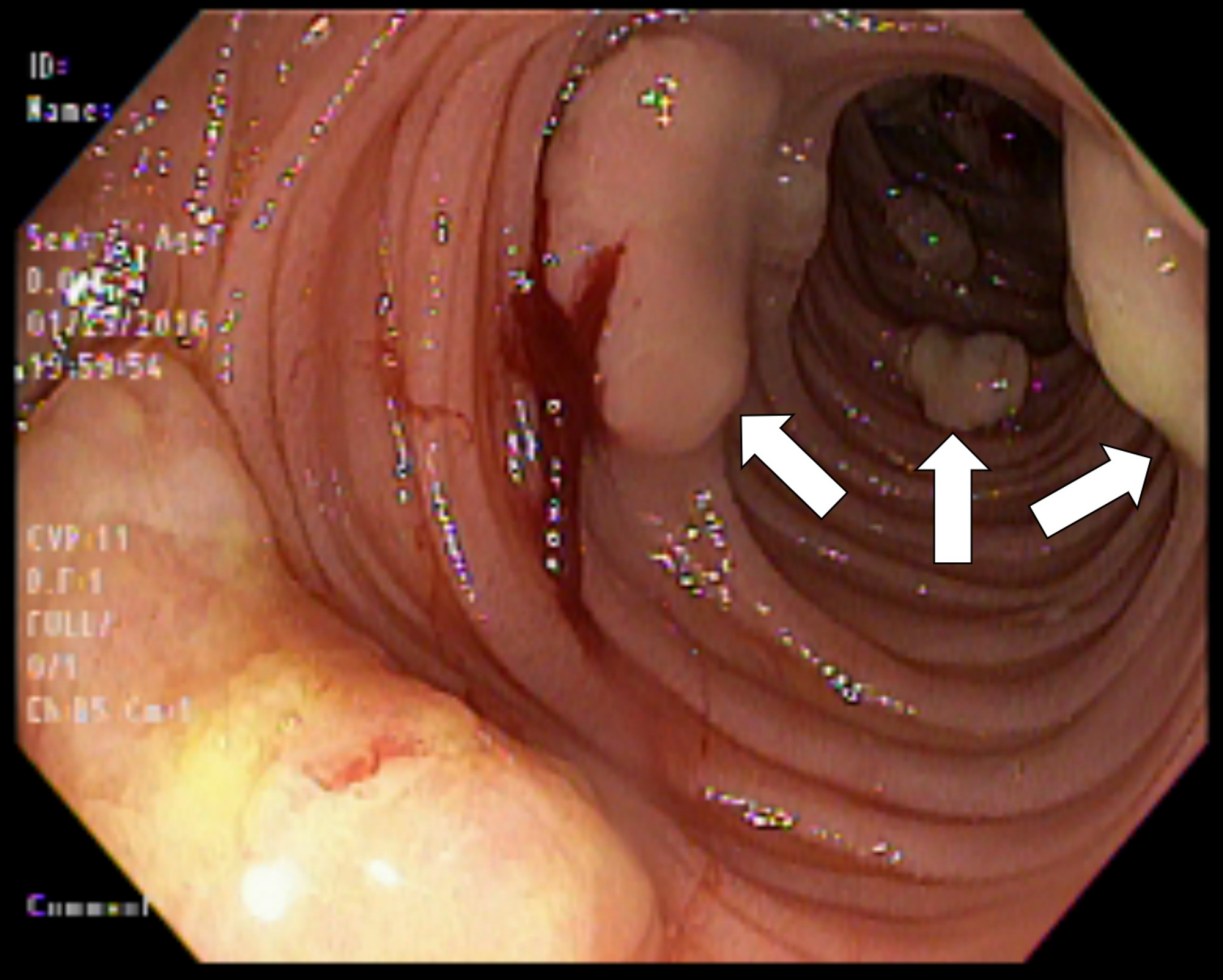
Friable nodules seen in duodenum.

Also, we noted multiple hard, friable nodules ranging in size from 5 mm to 2 cm starting at the second portion of the duodenum and extending into the visualized jejunum (Figures 1-2); multiple biopsies were obtained.

**Figure 3:**
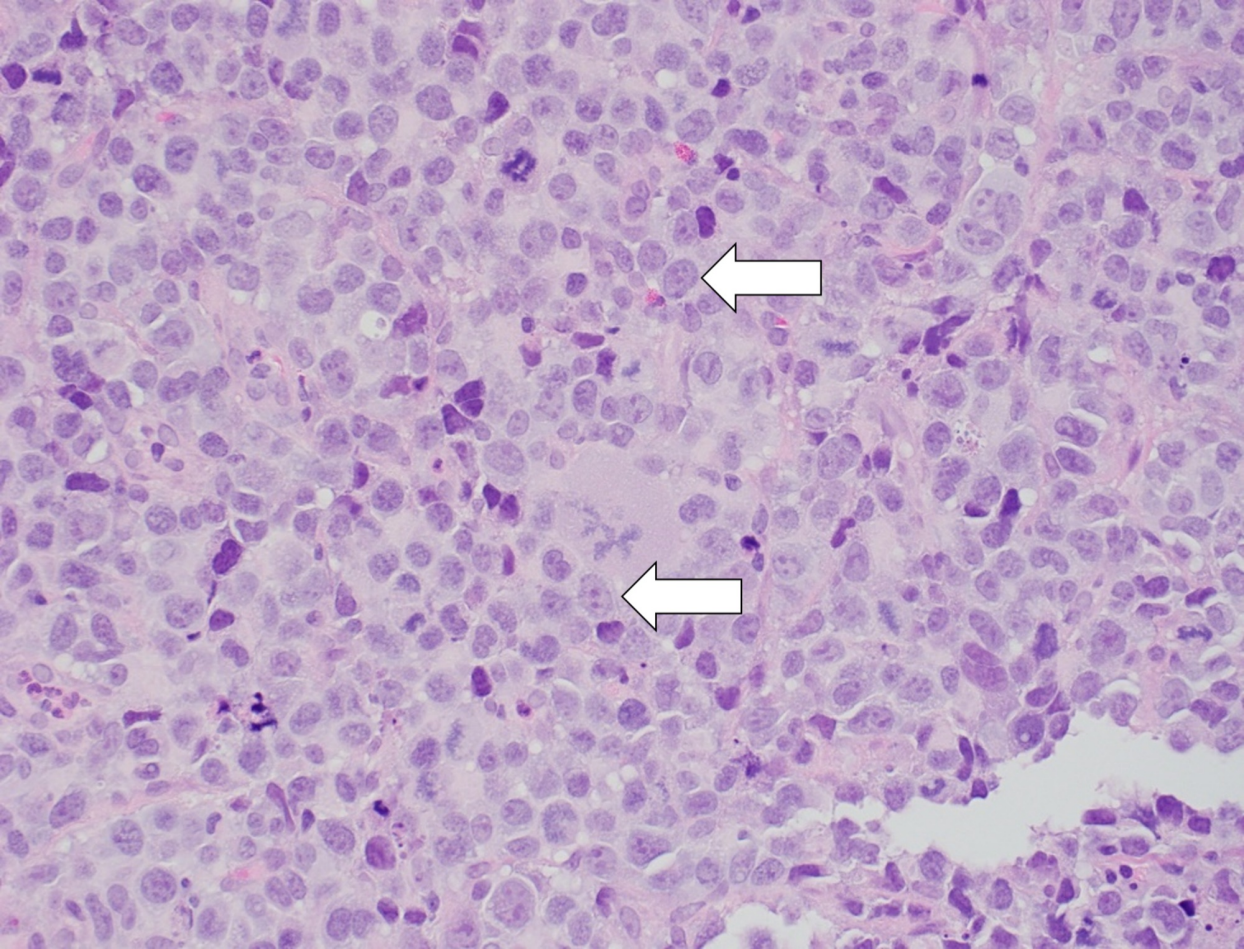
Microscopy image showing malignant lymphocytes are very large with moderately abundant cytoplasm, and the nuclei are round to ovoid with prominent nucleoli and occasional mitoses (hematoxylin and eosin stain, 400x).

The biopsy from the colon revealed colonic mucosa with mild stromal edema and focal lymphoid aggregate. The terminal ileum biopsy revealed small intestinal mucosa with preserved villous architecture. The small intestine, jejunum biopsy was significant for high-grade B-cell lymphoma showing small intestinal mucosa with submucosal large malignant lymphocytes with a moderately abundant cytoplasm (Figure 3).

**Figure 4:**
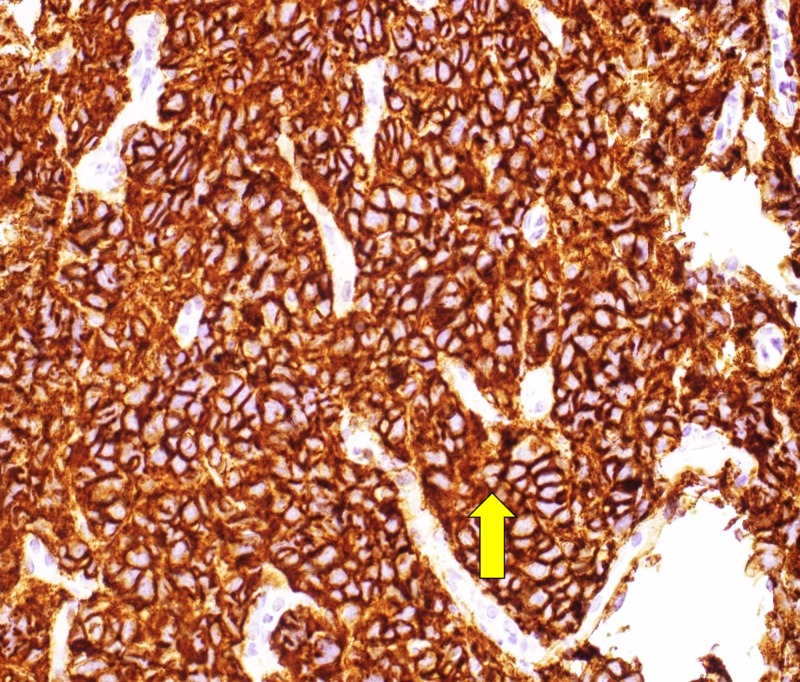
Microscopy image showing malignant lymphocytes positive for CD20 antibody (CD20 immunohistochemistry, 400x).

Immunohistochemistry results showed that cells were positive for CD20 (Figure 4) and CD3.

**Figure 5:**
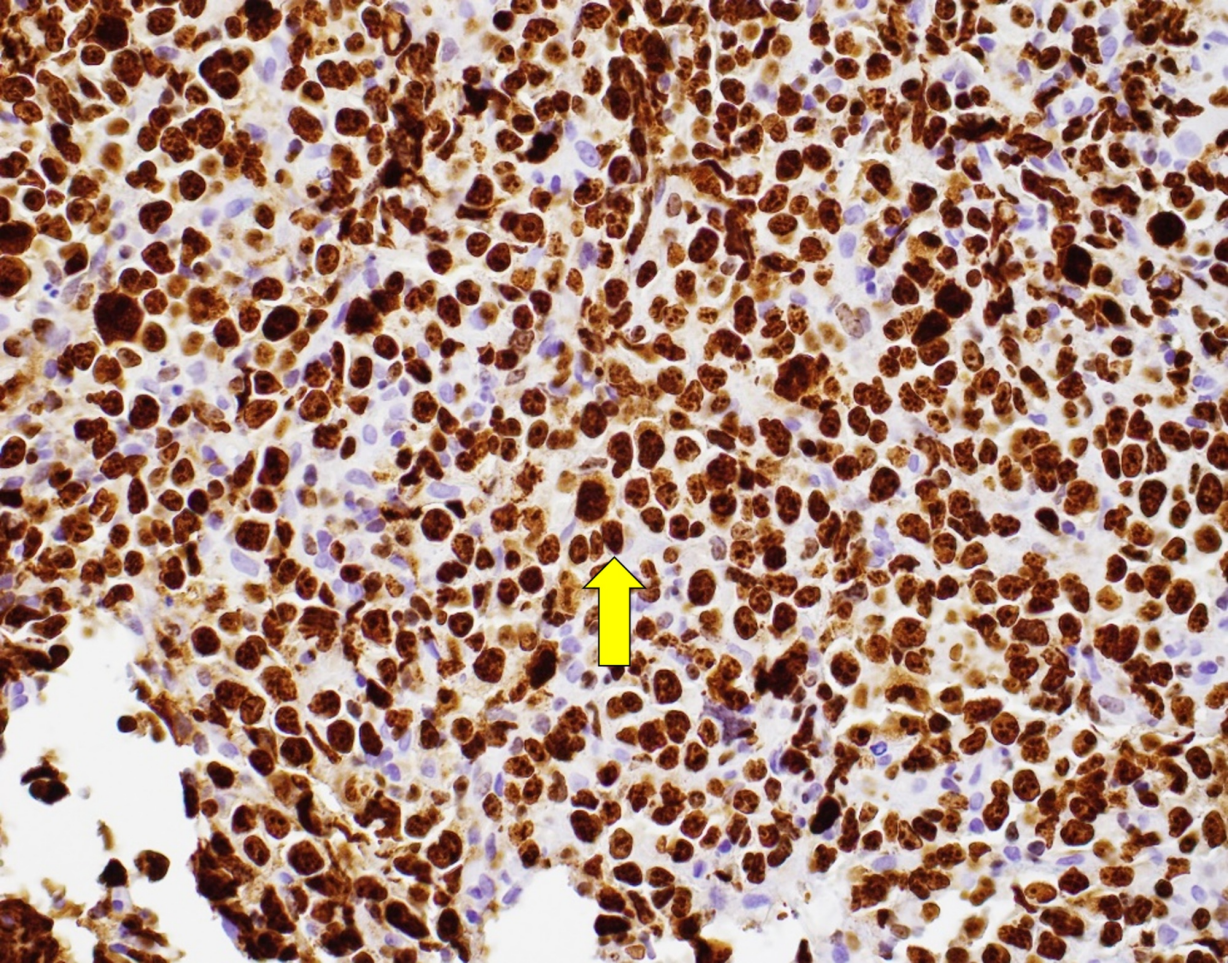
Ki67; proliferative index is very high (>90%). Arrow pointing towards cell taking up high levels of stain.

The Ki-67 stain was positive showing large atypical cells (Figure 5).

Stomach, antrum, and body biopsies showed negative immunostain for *Helicobacter pylori* and some evidence of mild chronic inflammation. A bone marrow biopsy, clot, and aspirate showed small lymphoid aggregate and hemosiderosis with no evidence of lymphoma.

In the context of the patient’s AIDS, our differential diagnoses were medication-induced pancreatitis, chronic pancreatitis, cholecystitis, and peptic ulcer disease. Regarding endobronchial findings, the differential would consist of familial adenomatous polyposis and its variants such as Turcot’s and Gardner syndromes, as well as mucosa-associated lymphoid tissue (MALT) lymphoma. The patient was started on chemotherapy with dose-adjusted rituximab with etoposide, prednisone, vincristine, cyclophosphamide, and doxorubicin (DaRR-EPOCH).

The patient was readmitted to our facility with recurrent seizures. Records from the outside hospital indicated she was recently diagnosed with toxoplasmosis after a magnetic resonance imaging (MRI) scan showed multiple new irregular predominantly peripheral enhancing lesions. She was receiving treatment. However, her care team recommended she undergo further testing which she refused. She signed out against medical advice.

## Discussion

Gastrointestinal lymphoma is a rare entity and, in most cases, is secondary to extranodal involvement of Non-Hodgkin’s lymphoma [4]. Primary gastrointestinal lymphomas are uncommon and constitute 5%-10% of all gastrointestinal tumors [5]. The most commonly involved sites in descending order are stomach (60%-75%), small intestine, and ileocecal region [6-7]. In the small intestine, the most commonly affected region is ileum followed by the jejunum, duodenum (6%-8%), and other regions [8].

A 10-year retrospective analysis conducted in the United States reported a 1.7:1 male-to-female predominance for intestinal DLBCL [9]. A Japanese study on 40 primary gastrointestinal tumors of the small and large intestines showed maximal incidence in patients aged 40-60 years [4]. The histopathological review suggested most primary gastrointestinal lymphomas have a B-cell lineage with DLBCL being the most common type [1, 5, 10]. Some lymphomas are associated with specific sites such as MALT lymphoma which is mostly found in the stomach and often has a *H. pylori* infection risk factor.

Pathogenesis of primary gastrointestinal DLBCL is very complex, involving genetic changes with aberrant gene and protein expression. It involves complex interactions of host immune cells with foreign antigens leading to activation of signaling pathways, which results in the proliferation of lymphoma cells.

DLBCL originates from germinal center B-cells or post-germinal B-cells, most of which express the B-cell lymphoma 6 (BCL6) gene which has also been implicated in the pathogenesis of DLBCL [11]. BCL6 negatively regulates its expression by binding to BCL6 a promoter. In DLBCL, BCL6 has a persistent expression or overexpression caused by chromosomal translocation leading to lymphomagenesis via various steps which include the inhibition of cell cycle arrest, modulation of apoptosis with down-regulation of target genes including the TP53 tumor suppressor gene, rearrangement of the MYC gene, and constitutive activation of signaling pathways [12]. Other mechanisms involve but are not limited to somatic hypermutation and altered tumor cell motility.

Human immunodeficiency virus, causing a depletion of CD4+ T lymphocytes leading to AIDS, is a known risk factor for lymphoma [13]. Non-Hodgkin’s lymphoma is the second most common type of malignancy in HIV patients after Kaposi sarcoma [5]. The pathogenesis of DLBCL in HIV is less understood. The culprit may be immune dysregulation in the setting of T-cell immunodeficiency and chronic B-cell stimulation which leads to a loss of control of viruses, most importantly, Epstein-Barr virus [14].

There are various subtypes of DLBCL based on histopathological findings. Typical features include complete disruption of normal architecture by atypical lymphoid cells. Tumor cells resemble normal lymphoblasts with large prominent nucleoli with abundant cytoplasm [15]. DLBCL expresses mostly all B-cell antigens including CD19, CD20, CD22, and CD45 [16]. Immunohistochemical evaluation, in most cases, shows a high proliferative index with a Ki-67 index of >80% [8].

With the involvement of the small intestine, symptoms are nonspecific. The most common symptoms are abdominal pain, nausea, vomiting, weight loss, gastrointestinal bleeding, and, in rare cases, intussusceptions and perforations [1, 3]. Diagnosis is made via biopsy. Currently, endoscopy is used to identify suspicious lesions preceded by radiographical evaluation. In some rare cases, the condition can present as multiple lymphomatous polyposes [4].

Some radiological features (e.g., significant lymphadenopathy and maintenance of the fat plane) are suggestive of lymphoma. However, a diagnosis cannot be wholly established on these radiological features [1].

Once a diagnosis is made, Ann Arbor staging with Musshoff modification is used to stage gastrointestinal lymphoma. Various modalities are used for staging purposes, including CT, MRI, positron emission tomography scanning, endoscopic ultrasound or molecular markers. Bone marrow biopsy is performed in the event of lymphoma cell involvement and for monitoring the response to treatment.

No guidelines exist for the treatment of DLBCL of the small intestine. We suggest a possible preferential management based on our review of the data with chemotherapy as the first-line therapy, given the overall response to chemotherapy is better in gastrointestinal B-cell lymphoma when compared with T-cell subtype. For gastrointestinal lymphoma, surgery followed by adjuvant chemotherapy was the preferred treatment modality based on systemic reviews. However, this preference has been a point of recent debate [17-18]. In a systemic review, several studies showed improved survival with surgery [17]. These reviews lack stratification and are based on individual author experiences. Chemotherapy is preferred by many physicians, particularly when treating patients with a high-grade tumor such as DLBCL [4]. With the advent of rituximab (a chimeric monoclonal antibody against the protein CD20), use of DaRR-EPOCH as the first-line therapy in high-grade B-cell lymphoma can be beneficial [19]. However, rituximab use has raised concerns about a higher incidence of neutropenic infections.

Historically, in patients with HIV, chemotherapy combined with antiretroviral therapy remains the first step in the management of aggressive lymphomas [5]. Radiotherapy, as a monotreatment modality, has not been shown to be beneficial in the treatment of DLBCL of the small intestine [20].

The international prognosis index (IPI) is used for prognosis in DLBCL. Poor prognostic factors include a patient age of >60 years, high serum levels of lactic acid dehydrogenase, Eastern Cooperative Oncology Group performance status ≥2, clinical staging of II or IV, and more than one extranodal disease [1]. Prognosis with use of chemotherapy has been favorable, and IPI, as a prognosis tool, has to be modified.

## Conclusions

In the evaluation of a patient with HIV reporting abdominal pain and vomiting, we should consider primary gastrointestinal lymphoma as part of the differential diagnosis. Chemotherapy combined with antiretroviral therapy is the cornerstone for management of high-grade DLBCL in patients with HIV.
